# Conditions Simulating Renal Calculus II

**Published:** 1892-07-30

**Authors:** 


### SURGERY.

### CONDITIONS SIMULATING RENAL CALCULUS.—II.

In a recent paper we described some conditions which simulated renal calculus. These were tuberculous disease of the kidney and malignant growths. In the present paper we propose to consider some other conditions which give rise to symptoms of stone, often so much so that exploratory operation has to be undertaken in order to decide the nature of the disease. But before doing so it may be well to say a word or two about this exploratory operation. We now know, not only that the kidney may be punctured all over in search after a stone, and the pelvis of the kidney opened and explored, but that exploratory wounds through the renal cortex heal readily enough, and may be made without serious hæmorrhage. Some surgeons unite them with cat-gut, and this checks the bleeding at the same time that it closes the wound. Until late years surgeons were content merely to explore the kidney *in situ*, but now Mr. Henry Morris always brings the kidney out on the loin, and through an incision in the renal cortex gets one finger well inside and the other out, and then between the two fingers explores every part of the organ, so that he feels sure no stone can escape detection. The risk of exploration in this way is very small. We used to be told that we must not drain out the kidney, that such traction on the vessels was to be avoided, but Mr. Morris does not seem to have found any ill-consequences result from it. Mr. Morris has explored the kidney in 28 cases without finding stone, but in 21 disease of some kind in and around the kidney was discovered, and that in 15 cases restoration to active life, with complete recovery in 14, was the result. In no case did any ill-consequences result from the exploration, and many cases that were cured were rescued from a life of incapacity from suffering. Under these circumstances Morris rightly urges physicians to recommend exploration of the kidney in doubtful cases. And now let us consider some of the conditions other than tubercular kidney and renal new growths, which may so simulate stone, that sometimes the diagnosis can only be cleared up by exploration, and first we will take injuries of the back. Injuries of the back—twists, &c.—may dislodge a calculus out of its resting-place where it was so fixed that it could not produce irritation from movement, and set it in the pelvis, where its movem ent would cause symptom before absent, and thus in this way start symptoms really due to renal calculus. But when no renal calculus is there, sprains of the back may cause symptoms of renal calculus—pain and hæmaturia, long after the injury—in two ways, either by causing thickening and condensation of the tissue around the kidney, which compresses the kidney, or actual laceration or bruising of the kidney leading to the development of induration and sclerosis in the kidney. At any rate, these are the conditions Morris found, and the shelling out of the kidney from the indurated surrounding tissue in the one case, and simply exploring the organ in the other, were followed by complete relief; why, in the latter case, it is difficult to see.

Mr. Morris has pointed out that a stone impacted in the ureter might cause the same symptoms as renal calculus.* He operated upon a patient for renal calculus, and only found a sacculated calyx in its upper part, surrounded by a very thin layer of renal tissue. It was just such a cavity as might contain a stone, but no stone was found in it, or in any other part of the kidney, though the organ was examined in the very thorough manner described before. Soon after the wound had healed he passed a bean-sized calculus by the urethra which Morris considers was blocking the ureter at its lower end when he operated on the kidney, and had given rise to symptoms of calculus. But why was not the stone dislodged into the ureter at the time the kidney was manipulated; this seems quite as probable, and seems to emphasize the need to pass a long probe down the ureter if possible, and make sure no stone is there.

In cases of suspected calculus, it is always wise to sound the bladder before exploring the kidney. Mr. Morris has on three occasions removed large calculi from the bladder by lithotrity in patients who were sent for nephrolithotomy, in both of these cases the bladder had been previously sounded, but the stones were so coated with ropy muco-pus that they were only discovered with the sound after thoroughly washing out the bladder. In the third case the bladder had not been sounded, but the case was diagnosed as renal calculus by a "distinguished provincial physician." In cases of any doubt Morris always also examines the prostrate and vesiculae seminales *per rectum*.

* *American Journal of Medical Sciences*, Oct. 1884.

Movable kidney is another condition which may simulate renal stone. In such cases though no stone is found on exploration we can perform nephrorraphy and relieve the condition in this way. In one case in which Mr. Morris operated, "with the finger kept fixed in the operation wound the kidney could be passed in its entirety above, below, and to the inner and outer side of the finger tips."

Disease in connection with the gastro-intestinal tract may simulate renal stone. In the cases explored for stone by Mr. Morris one turned out to be gastric ulcer and another disease around the cœcum.

Both Morris, Jackson, and Wright have called attention to the way in which spinal caries may simulate renal calculus. Mr. G. A. Wright has recorded a case in which an abscess due to spinal disease so pressed on the kidney as to cause symptoms of renal stone, and in this case there was no excurvation or tenderness of the spine to assist the diagnosis.*

* *Med. Chron.* No. vi., p. 642.

